# Morphological, Mechanical, and Antimicrobial Properties of PBAT/Poly(methyl methacrylate-*co*-maleic anhydride)–SiO_2_ Composite Films for Food Packaging Applications

**DOI:** 10.3390/polym15010101

**Published:** 2022-12-26

**Authors:** Raja Venkatesan, Krishnapandi Alagumalai, Chaitany Jayprakash Raorane, Vinit Raj, Divya Shastri, Seong-Cheol Kim

**Affiliations:** 1 School of Chemical Engineering, Yeungnam University, Gyeongsan 38541, Republic of Korea; 2 School of Pharmacy, Yeungnam University, Gyeongsan 38541, Republic of Korea

**Keywords:** PBAT, P(MMA-*co*-MA) copolymer, SiO_2_ nanoparticles, PBAT/P(MMA-*co*-MA)–SiO_2_ composites, mechanical strength, antimicrobial activity, food packaging

## Abstract

A poly(methyl methacrylate-*co*-maleic anhydride) P(MMA-*co*-MA) copolymer was synthesized via radical polymerization. The synthesized P(MMA-*co*-MA) copolymer was identified by ^1^H- and ^13^C-nuclear magnetic resonance spectroscopy (^1^H-NMR), (^13^C-NMR), Fourier-transform infrared (FTIR) spectroscopy, X-ray diffraction (XRD), scanning electron microscopy (SEM), and transmission electron microscopy (TEM). The poly(butylene adipate-*co*-terephthalate) (PBAT)/P(MMA-*co*-MA)–SiO_2_ composites were developed using a solution-casting method. The PBAT to P(MMA-*co*-MA) weight ratio was kept at 70:30, while the weight percentage of SiO_2_ nanoparticles (NPs) was varied from 0.0 to 5.0 wt.%. SiO_2_ was used for PBAT/P(MMA-*co*-MA) to solve the compatibility between PBAT and the P(MMA-*co*-MA) matrix. The PBAT/P(MMA-*co*-MA)–SiO_2_ composites were characterized by studied FTIR spectroscopy, XRD, SEM, and TEM. A comparison of the composite film PBAT/P(MMA-*co*-MA)–SiO_2_ (PBMS-3) with the virgin PBAT and P(MMA-*co*-MA) film revealed its good tensile strength (19.81 MPa). The WVTR and OTR for the PBAT/P(MMA-*co*-MA)–SiO_2_ composites were much smaller than for PBAT/P(MMA-*co*-MA). The PBAT/P(MMA-*co*-MA)–SiO_2_ WVTR and OTR values of the composites were 318.9 ± 2.0 (cc m^−2^ per 24 h) and 26.3 ± 2.5 (g m^−2^ per 24 h). The hydrophobicity of the PBAT/P(MMA-*co*-MA) blend and PBAT/P(MMA-*co*-MA)–SiO_2_ composites was strengthened by the introduction of SiO_2_, as measured by the water contact angle. The PBAT/P(MMA-*co*-MA)–SiO_2_ composite films showed excellent antimicrobial activity against the food-pathogenic bacteria *E. coli* and *S. aureus* from the area of inhibition. Overall, the improved packaging characteristics, such as flexibility, tensile strength, low O_2_ and H_2_O transmission rate, and good antimicrobial activities, give the PBAT/P(MMA-*co*-MA)–SiO_2_ composite film potential for use in food packaging applications.

## 1. Introduction

The advances in food packaging materials in recent decades have attracted considerable attention because of the increasing need for effectively packaged foods with an extended shelf life [1]. The active film can offer extra safety features in addition to the usual barrier characteristics [2,3]. The antioxidant and antimicrobial activities are significant because they help alleviate concerns with food quality, such as degradation, rancidity, and coloration. An active food packaging material should fulfill a list of needs, including having sufficient physical and mechanical properties, a high barrier, a high level of barrier performance, safe for human health, and no harmful impact on the environment [4]. Furthermore, a straightforward, reproducible, and economical preparation method is essential for large-scale applications [5,6]. New production techniques that enhance the safety of food packaging are available. The manufacture of food packaging must ensure that the packaging volume and weight are kept to a minimum to maintain the required stability, hygienic conditions, and consumer acceptance. However, there are significant obstacles to using biodegradable food packaging in markets. Technologies that enhance the oxygen and water barrier of biodegradable polymer systems are ideal for biodegradable packaging in daily life. In recent times, notable potential improvements have been adopted, showing promise in fabricating high oxygen/water vapor-permeable biodegradable materials for food packaging.

Radical polymerization is the primary method for synthesizing poly(methyl methacrylate-*co*-maleic anhydride) P(MMA-*co*-MA) [7]. Pre-polymerization and cast polymerization are the two steps that are always involved in polymerization [8]. Furthermore, P(MMA-*co*-MA) copolymer can be used in ring-opening reactions to form cube-shaped materials that can be used in various applications, such as electrolytes for batteries, surfactants and emulsified solutions, and antiwear additives [9,10]. It is often used to fabricate composite materials that transfer heat easily because of its excellent properties [11,12,13]. P(MMA-*co*-MA) is the focus of the current research, which shows a relationship with particle size [14], flame retardants [15], inorganic nanoparticles [16], bio-additives [17], chemical modification [18], and polymerization conditions [19]. On the other hand, the brittleness and poor mechanical characteristics of P(MMA-*co*-MA) constitute a significant impediment to its broad application. Some research has been conducted to develop P(MMA-*co*-MA) composites with reinforcement materials, such as polymers, chitin nanofibers, and cellulose, to increase the mechanical and thermal properties and solve the issues [11]. Nevertheless, there are still a few disadvantages. For example, it cannot substantially enhance the mechanical properties of P(MMA-*co*-MA) materials, and using nanofillers can increase the cost of P(MMA-*co*-MA) composites, which is another impediment to scaling up production. Impurities cause opacity and the use is confined because of the hydrolysis reaction of the maleic anhydride groups [20,21]. Maleic anhydride (MA) and methyl methacrylate (MMA) were copolymerized. Other components may alter P(MMA-*co*-MA) to circumvent these limitations [12,22].

As a transparent thermoplastic, lightweight, or shatter-resistant replacement for glassware, poly(butylene adipate-*co*-terephthalate) (PBAT) was blended with P(MMA-*co*-MA). It is non-toxic and contains a strong hydrogen bonding character. It can also be used as a casting polymer in inks and coatings. Owing to its plasticizing property [9], PBAT increases the flexibility and barrier characteristics of the produced composite film, which are essential for packaging applications [23,24]. The most notable biocompatibility polymers in the thermoplastic materials group, PBAT, are synthesized via reactions among the monomers adipic acid, terephthalic acid, and 1,4-butanediol [25]. This material is attractive because of its excellent physical features, high elongation at break, and amorphous structure. In addition, it can be obtained using eco-friendly and renewable technologies [26,27]. The capability to produce scaffolding structures enables PBAT to be used in tissue engineering to reconstruct or replace tissue (such as bone, cartilage, and blood vessels). On the other hand, reinforcing fillers must strengthen the PBAT matrix’s performance and enhance its mechanical and thermal properties for broader use. Countless loadings in the PBAT matrices have been studied to improve the physical, thermal, permeability, and characteristics of the finished goods.

Nano-silica is used in combination with PBAT/P(MMA-*co*-MA) blends as a nanofiller to improve the characteristics of materials owing to its low cost, high reproducibility, and suitability for large-scale production [28]. SiO_2_ nanoparticle (NP) synthesis is a topic of interest in basic and applied research. Other variables include the particle-size suitability for multiple products. The last few years have seen a significant increase in interest in SiO_2_-filled composites. According to studies, composite materials loaded with SiO_2_ possess good thermal and mechanical characteristics [29,30]. Studies have been carried out on the SiO_2_ distribution in poly(methyl methacrylate) matrices [31,32], high-density polyethylene [33], and poly(ethylene oxide) [34]. PBAT is a thermoplastic compostable material commonly used to manufacture films, bottles, and fibers for different applications because of its outstanding mechanical and optical characteristics, susceptibility to fatigue and wear, and creep-fragmentation resistance [35,36]. Functional characteristics, such as antimicrobial activity, have been enhanced during the fabrication of PBAT-based composite films [37,38,39]. 

Recent research has been conducted on biomass or sustainable polymers reinforced with fillers and fabricated to use the solution casting method [40,41,42]. In this work, a series of PBAT/P(MMA-*co*-MA) composites with different contents was prepared by solution casting of PBAT/P(MMA-*co*-MA) blends and a SiO_2_ nanopowder [43]. SiO_2_ has a disk structure according to scanning electron microscopy (SEM). The excellent compatibility and interactions between SiO_2_ and PBAT mean that SiO_2_ NPs are dispersed uniformly in PBAT/P(MMA-*co*-MA) blends according to the shape of the film surface [44]. The tensile strength of the PBAT/P(MMA-*co*-MA) composites increased from 9.43 MPa for PBAT without reinforcement to 22.82 MPa with 5.0 wt.% of SiO_2_ NPs (PBMS-3). Thermogravimetric analysis (TGA) of the composite films of PBAT/P(MMA-*co*-MA)–SiO_2_ showed an increase in the weight loss temperature (%). The PBAT/P(MMA-*co*-MA)–SiO_2_ composite materials also have high hydrophobicity. The antimicrobial activity of SiO_2_-incorporated PBAT/P(MMA-*co*-MA) composites is remarkable because of the combined antimicrobial activity of SiO_2_ NPs against *S. aureus* and *E. coli*. As a result, composites composed of PBAT/P(MMA-*co*-MA)–SiO_2_ were developed and assessed for food packaging applications. 

## 2. Materials and Methods

### 2.1. Chemicals and Materials

Poly(butylene adipate-*co*-terephthalate) (PBAT) pellets were supplied by BASF. The melt flow index (MFI) (190 °C, 2.16 kg) was 3.3–6.6 g/10 min. A solution of methyl methacrylate (MMA) was purchased from Daejung Chemicals in Korea. Maleic anhydride (MA; 98%, Sigma-Aldrich, St. Louis, MO, USA) was recrystallized from chloroform and n-hexane. By crystallizing from methanol, the radical initiator of azobisisobutyronitrile (AIBN; 99%, Sigma–Aldrich, St. Louis, MO, USA) was purified. Tetraethoxysilane (TEOS) was received from Sigma–Aldrich in India. Daejung Chemicals in Korea supplied methyl benzene, ethyl acetate, ethanol, and N, N-dimethylformamide (DMF). All compounds were used as received. For the preparation of solutions and reactions, double-distilled water was used.

### 2.2. Synthesis of SiO_2_ Nanoparticles (NPs)

SiO_2_ NPs were synthesized using the methodology reported elsewhere [45]. Tetraethoxysilane (TEOS) was used as a silica source in the sol–gel technique to prepare SiO_2_ NPs. The reaction was rapidly stirred for 30 min at 150 °C with 7.4 mL TEOS and 80 mL methanol introduced. A white precipitate was heated for one hour at 350 °C in a tubular furnace to generate SiO_2_. The XRD, SEM, and TEM characterization data matched the information from the previous report [46].

### 2.3. Synthesis of Poly(methyl methacrylate-co-maleic anhydride)

**Figure 1:**
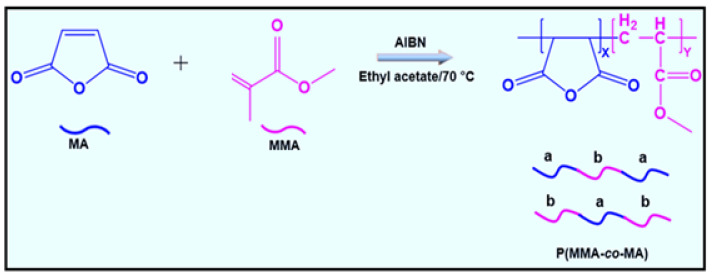
Synthetic routes of poly(methyl methacrylate-*co*-maleic anhydride) copolymer.

The methyl methacrylate and maleic anhydride monomers were prepared by a radical reaction to produce the P(MMA-*co*-MA) copolymer. Figure 1 depicts the synthesis of P(MMA-*co*-MA). Before use, the MMA monomer (1.50 g; 0.015 mM) underwent two reduced-pressure vacuum distillations at 35 °C. For 45 min, the polymerization reaction took place in a reactor with rapid mixing and a nitrogen atmosphere. The copolymers were synthesized using 3.43 g: 0.035 mM of (maleic anhydride) MA, ethyl acetate as the solvent, with AIBN as the radical polymerization initiator at 85 ± 1 °C. The method of synthesis used in this work can be found in the literature [12,47].

### 2.4. Purification of P(MMA-co-MA) Copolymer

The material was produced by mixing un-reacted MMA, MA, and P(MMA-*co*-MA) copolymer. After mixing the materials, the diluted solution was added to the diethyl ether in the precipitate for P(MMA-*co*-MA). A tetrahydrofuran/water (9/1 *v/v*) solution was used to dissolve the P(MMA-*co*-MA), resulting in a solution with a 2.0 10^−2^ g mL^−1^ specific concentration. The reaction was quenched when the solution became too viscous to be stirred (usually around 1 h). The reaction mixture was diluted in THF and precipitated in n-hexane to remove the unreacted initiator and monomer. This procedure was repeated three times to obtain the purified copolymer. The obtained P(MMA-*co*-MA) precipitate was dried under vacuum at 100 °C for 10 h.

### 2.5. Fabrication of PBAT/P(MMA-co-MA)–SiO_2_ Composites

PBAT and P(MMA-*co*-MA) were blended at a 70:30 ratio. This ratio was selected as it also generated a suitable blend of properties because PBAT was the target polymer and its properties needed to be enhanced. Solution casting methods were used to fabricate the PBAT/P(MMA-*co*-MA)–SiO_2_ composites [48]. A homogeneous solution was produced first by dissolving PBAT (1.0 g) in chloroform (50 mL) at room temperature for 12 h. Furthermore, 50 mL of P(MMA-*co*-MA) and chloroform (30 wt.% with respect to PBAT) were blended for one hour in an ultrasonic mixer. The produced solution was then transferred to a glass Petri dish after being sonicated, in which the chloroform solvent was allowed to dry completely. The chloroform was removed by scraping the PBAT/P(MMA-*co*-MA) composites off the Petri plate and drying them at 60 °C under vacuum for eight hours. The PBAT/P(MMA-*co*-MA) blends were stored in an airtight container for future analysis. In contrast, a PBAT and P(MMA-*co*-MA) film was prepared by adding chloroform solvent and casting it onto a glass Petri dish after mixing for an hour. Removing the dry films allowed samples to be kept in a desiccator at 23 °C until tested. On the premise of the wt.% of SiO_2_ it includes, the PBAT/P(MMA-*co*-MA) blend film was given the designations PBMS-1, PBMS-2, and PBMS-3. Table 1 lists the data collected.

### 2.6. Characterization

#### 2.6.1. Structural Characterization

The ^1^H-nuclear magnetic resonance (NMR) and ^13^C-NMR (600 MHz) spectra were recorded on a Bruker instrument (OXFORD, AS600, USA) using deuterated chloroform (CDCl3) as the solvent and tetramethyl silane as the reference. The Fourier-transform infrared (FTIR, Perkin–Elmer Spectrum Two) spectra were obtained to produce the attenuated total reflection (ATR)-FTIR spectra in a 4000–400 cm^−1^ wavenumber range, and X-ray diffraction (XRD, Rigaku, PANALYTICAL) was performed at a scan rate of 0.50 min^−1^ over a scan range of 10 to 80° 2θ. 

#### 2.6.2. Morphological Studies

The surface morphology of clean PBAT, P(MMA-*co*-MA), and its composites was examined by SEM (JEOL 6400, Tokyo, Japan). TEM (JEOL, JEM-2100, Japan) was employed to investigate the inner microstructure of PBAT/P(MMA-*co*-MA) and composites. A small amount of the samples was sonicated in ethanol for descriptive purposes, and a drop of it was cast in a 300-mesh copper grid with carbon coating for electromagnetic measurements. 

#### 2.6.3. Thermal Characterization

Thermogravimetric–differential thermal analysis (TG–DTA, TA Instruments, SDT Q6000) was used to evaluate the thermal stability. Under a N_2_ atmosphere, all TG–DTA was conducted from 50 °C to 700 °C at a scanning rate of 20 °C/min (nitrogen flow rate was 60 mL min^−1^). Differential scanning calorimetry (DSC, A Instruments, DSC Q200). was performed using 5.0 mg of the PBAT/P(MMA-*co*-MA)–SiO_2_ composite samples. The samples were heated from room temperature to 300 °C in a N_2_ atmosphere at a flow rate of 50 mL per minute. The thermal history in the case of PBAT was removed by heating the sample to 180 °C and holding it at that temperature for two minutes. The sample was then cooled to 50 °C and heated to 180 °C again. The heating rate used for all DSC runs was 20 °C/min.

#### 2.6.4. Mechanical Strength Measurements

The tensile strength of PBAT/P(MMA-*co*-MA) and its composites was evaluated on a universal testing machine (3345, Instron, Norwood, MA, USA) in accordance with ASTM-D882 at 23 °C and 50% RH. The PBAT composite samples used for the evaluation had the following dimensions: 50 × 20 mm, gauge length of 30 mm, and speed of 20 mm/min. Five tensile-strength samples were tested and the average result was calculated. In MPa, the maximum tensile strength was specified. The digital thickness measurement instrument (Mitutoyo micrometer, Japan) determined the film thickness to the closest 0.001 mm. The mean of at least five locations was used to determine the values. The mechanical and physical characteristics of the materials were measured to use the estimated values. 

#### 2.6.5. Barrier Properties

The OTR measurements for the PBAT/P(MMA-*co*-MA) and its composites were evaluated using a *Noselab* (ATS, Concorezzo, Italy) at 23 °C and 50% RH according to the ASTM D3985 standard method. The machine had a one-atm pressure. Five different locations on the composite samples were measured and the average value was used. The specimen was processed at room temperature. A *Lyssy L80-5000* was used to measure the WVTR values of the PBAT/P(MMA-*co*-MA) blends and PBAT/P(MMA-*co*-MA)–SiO_2_ composites in accordance with ASTM F1249-90 under the conditions of 100% RH at 23 °C. Five repeats of the test were carried out prior to calculating the average result. 

#### 2.6.6. Water Contact Angle Measurements

Using a contact angle meter (Dataphysics Instruments, OCA-20, Filderstadt, Germany), the sessile drop method, at 23 °C, and 50% RH, the water contact angle of the PBAT and its composites was studied. A 1µL droplet of water was placed on PBAT and the composite surface of the films and a droplet image was taken within 5 s to measure the contact angle. The mean value was calculated after the surface tension measurements were performed at five different locations on the film. The experimental result has a ±1° confidence interval.

#### 2.6.7. Antimicrobial Activities

The antimicrobial activities of PBAT/P(MMA-*co*-MA) with SiO_2_ NPs were tested using the zone-of-inhibition technique. In compliance with ASTM E 2149-01, the composites were tested in advancing contact conditions against Gram-negative (*E. coli*) and Gram-positive (*S. aureus*) microorganisms. To prepare a broth, the beef extract (1.0 g) and peptone were mixed in 100 mL of water (2.0 g). A shaking incubator at 40 °C and 200 rpm was applied to cultivate the solution for 24 h. The 0.90% sterile NaCl aqueous solution was used to dilute the microorganism cell suspension by a frequency of 10^6^ for *E. coli* and *S. aureus*.

#### 2.6.8. Statistical Analysis

*ANOVA* in SPSS 21 was used (IBM, New York, NY, USA) to determine the statistical significance of each result. The data are given as the mean ± standard deviation. Statis- tical differences were analyzed using a one-way analysis of variance, and a value of *p* < 0.05 was considered significant. 

## 3. Results and Discussion

### 3.1. Characterization of SiO_2_ Nanoparticles

**Figure 2:**
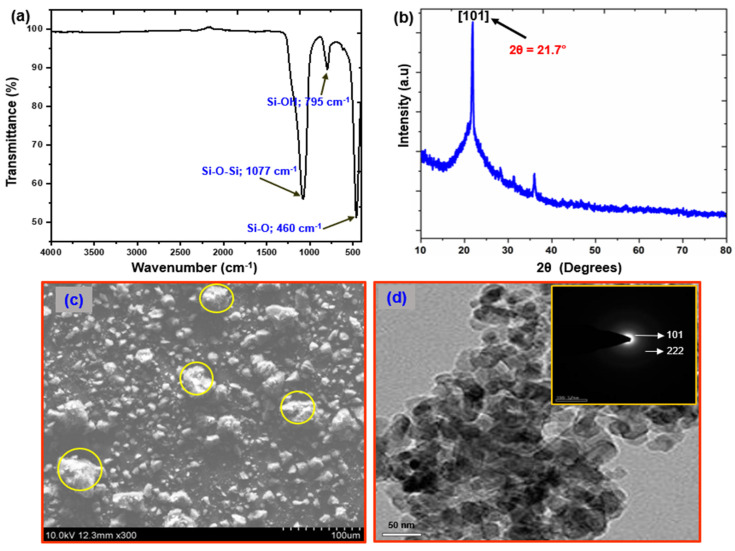
Structural and morphological characterization of SiO_2_ nanoparticles: (**a**) FTIR spectrum; (**b**) XRD pattern; (**c**) SEM; and (**d**) TEM images (inset in d is the SAED pattern).

FTIR spectroscopy of the synthesized SiO_2_ NPs was conducted (Figure 2a). The two major peaks at 1077 and 460 cm^−1^ suggest the presence of SiO_2_ NPs as asymmetric and symmetric Si-O-Si modes, respectively [49]. The Si–O stretching vibration for surface Si–OH groups was observed at 795 cm^−1^. Rahman et al. suggested that the Si–O–Si and Si–OH groups on SiO_2_ NPs help compensate for the silica network [50]. Figure 2b shows the XRD pattern of the SiO_2_ NPs. Two reflection peaks were observed at 21.7° 2θ, which are at the scattering angle from the (101) lattice planes, showing that the SiO_2_ is crystalline [51]. The Scherrer equation showed that the size of crystalline SiO_2_ NPs was 20 nm. SEM and TEM were used to characterize the SiO_2_ NP structure. SEM revealed the surface of SiO_2_ (Figure 2c). The SiO_2_ nanoparticles had a mean diameter of 20 nm and a uniform morphology [52]. SiO_2_ ranged in size from 20 to 50 nm, and TEM showed that it was almost spherical (Figure 2d). Figure 2d’s inset shows the selected area electron diffraction (SAED) pattern of synthesized SiO_2_. Only a few diffraction spots scattered in a circle can be seen when electron diffraction is conducted on a small proportion of crystals. The (101) and (222) planes of the face-centered cubic silica structure were fitted by the patterns. It is also feasible to determine the crystalline behavior from the SAED image.

### 3.2. Characterization of P(MMA-co-MA) Copolymer

**Figure 3:**
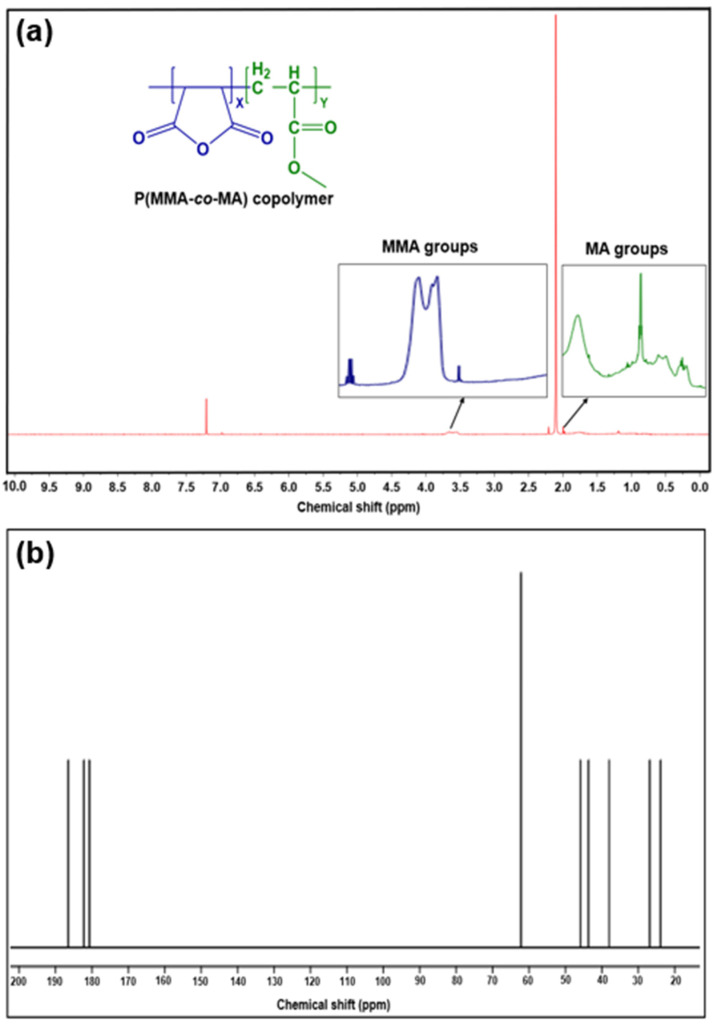
(**a**) ^1^H-NMR spectrum; (**b**) ^13^C-NMR spectrum of the P(MMA-*co*-MA) copolymer.

Figure 3a presents the P(MMA-*co*-MA) and ^1^H-NMR spectra. The chemical shifts at 5.55 ppm and 6.09 ppm in the copolymers were produced by the double-bond proton of the MMA molecule. The copolymer MA units, which have methylene protons, are responsible for the absorption peaks at 6.35 and 7.25 ppm. The integrated areas at 0.65 and 1.45 ppm reflect the methyl protons of the MMA units and the integrated areas at 1.65 and 2.07 ppm reflect the methylene protons [53]. The methyl protons of –COOCH_3_ are essential for the absorption peaks at 3.42 and 3.87 ppm. Figure 3b displays the ^13^C-NMR spectrum of the P(MMA-*co*-MA) copolymer. From 180.7 to 182.2 ppm, the carbonyl carbon (>C=O) signals of both MA and MMA units could be seen. The back and side chain of the P(MMA-*co*-MA) copolymer show aliphatic carbon resonance in a spectral region from 26.9 to 38.0. In 62.2 (^6^CH_2_), 43.7 (^4^CH_2_), and 38.2, the side-chain-ring methylene carbon signal was assigned (^5^CH_2_). The methyl carbon of MMA is shown by the signal at 45.9 (^11^CH_3_). The carbon (^1^CH) and CH_2_ signals overlap.

**Figure 4:**
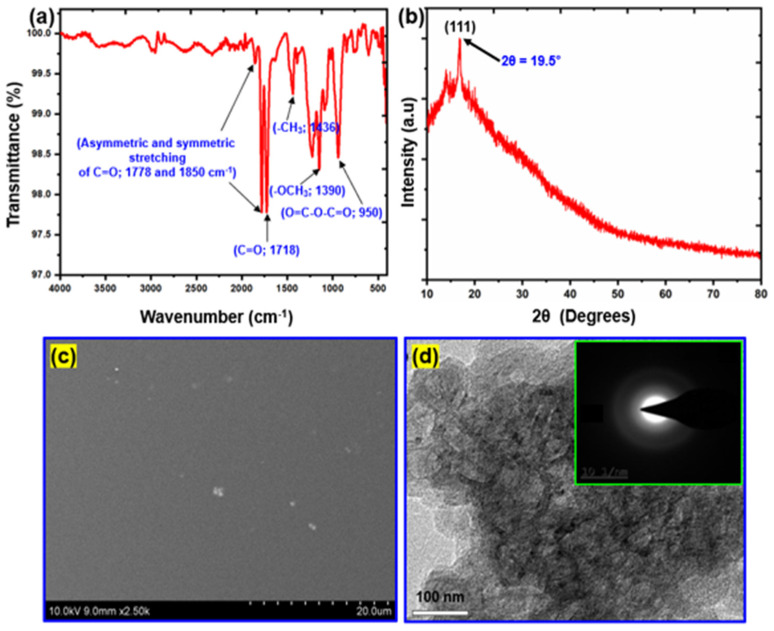
Structural and morphological characterizations of P(MMA-*co*-MA) copolymer: (**a**) FTIR spectrum; (**b**) XRD pattern; (**c**) SEM; and (**d**) TEM images (inset in d is the SAED pattern).

The FTIR spectra were used to study the P(MMA-*co*-MA) copolymer structures, as shown in Figure 4a. The asymmetric and symmetric stretching modes of C=O are represented by all of these anhydride units (1778 and 1850 cm^−1^), which are also found in P(MMA-*co*-MA). In addition, the distinct anhydride band (O=C–O–C=O; 950 cm^–1^) indicated that MA had been successfully introduced into the copolymers. The stretching vibration of the ester group (O-C=O; 1718 cm^−1^) in MMA was observed. The carbonyl groups of MA and MMA show three distinct peaks that closely follow one another, as indicated. Moreover, the maximum of the C–H stretching mode of MMA and MA (2948 cm^−1^), in addition to symmetric and asymmetry bend vibration of the -CH_3_ and -OCH_3_ bridges of the MMA unit (1436 and 1390 cm^−1^, accordingly), all indicated the existence of these monomer units within the resulting copolymers. MMA and MA performed a copolymerization reaction at 85 °C. Figure 4b shows the XRD patterns of synthetic P(MMA-*co*-MA) copolymer. XRD is the most outstanding method for smoothly finishing the material structure. The XRD pattern of P(MMA-*co*-MA) [54]. The characteristic broad peak at 19.5° 2θ, corresponding to the (111) plane, reveals the amorphous of P(MMA-*co*-MA). Figure 4c shows the SEM images of the synthetic P(MMA-*co*-MA) copolymer. Although the P(MMA-*co*-MA) SEM images are homogeneous and smooth, the P(MMA-*co*-MA) exhibits aggregated molecular structures. TEM was used to study the inner morphology of synthesized P(MMA-*co*-MA) copolymer. Figure 4d shows TEM images of the P(MMA-*co*-MA) matrix. The TEM images revealed a transparent portion of the images, representing the smooth and uniform structure of the P(MMA-*co*-MA). Figure 4(d)’s inset depicts the selected area electron diffraction (SAED) pattern of the synthesized P(MMA-*co*-MA) copolymer. The amorphous structure of the P(MMA-*co*-MA) copolymer is evident in its SAED image.

### 3.3. Characterization of PBAT/P(MMA-co-MA)–SiO_2_ Composites

**Figure 5:**
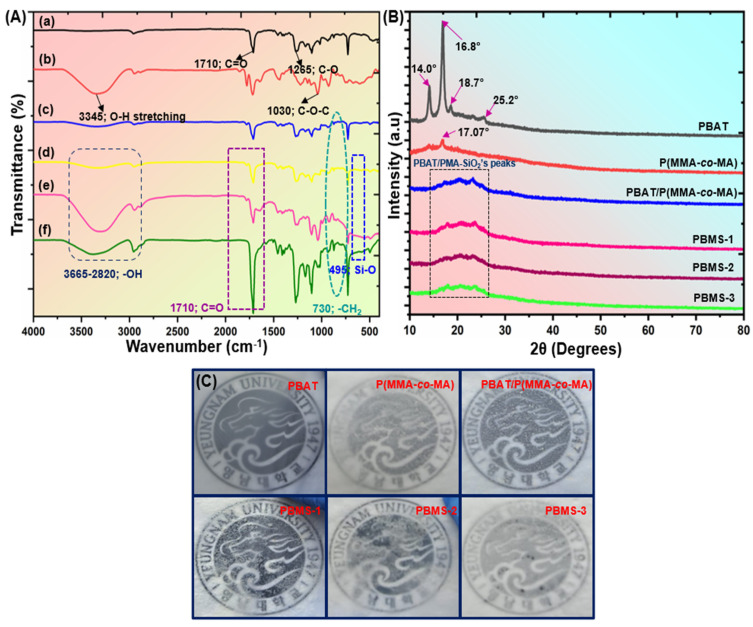
Structural characterization of PBAT/P(MMA-*co*-MA)–SiO_2_ composites: (**A**) ATR-FTIR spectra ((a) PBAT; (b) P(MMA-*co*-MA); (c) PBAT/P(MMA-*co*-MA) blends; (d) PBMS-1; (e) PSMS-2; (f) PBMS-3); (**B**) XRD patterns; (**C**) the appearance of the film.

Figure 5A shows the FT-IR spectra. The distinct peak at 2958 cm^−1^ was assigned to the stretching vibration of C–H groups [55,56]. The prominent peaks at 1780 and 1720 cm^−1^ were produced by the strong peak of the C=O group in the PBAT and P(MMA-*co*-MA). The peaks suggested the ester linkage (C–O) at 1270 and 1110 cm^−1^ of P(MMA-*co*-MA) copolymer. The bending vibration of the C–C can be ascribed to the peak at 1450 and 1390 cm^−1^, respectively. The vibration of the adjacent (CH_2_–) groups of the PBAT matrix was observed at 720 cm^−1^. In SiO_2_, the presence of silicon is confirmed by the peaks at 475 and 850 cm^−1^. In the PBAT/P(MMA-*co*-MA)–SiO_2_ ternary composites, the peaks for the PBAT/P(MMA-*co*-MA) blend film moved toward higher and lower wavenumbers. The shift toward a higher wavenumber is due to the link between the –COO group in PBAT and SiO_2_ through metal bonding. The FTIR results showed SiO_2_ with a suitable molecular attachment in the PBAT/P(MMA-*co*-MA) blends. The semi-crystalline characteristics of the PBAT/P(MMA-*co*-MA)–SiO_2_ composites were examined with XRD. Figure 5B shows XRD patterns of PBAT/P(MMA-*co*-MA)–SiO_2_ composites, and the prominent peaks were observed at 15.7°, 17.8°, 20.4°, 21.8°, 23.2°, 25.4°, and 28.0° 2θ, corresponding to lattice planes of (010), (020), (012), (110), (102), (210), and (101), respectively. PBAT/P(MMA-*co*-MA)–SiO_2_ composite films show two more peaks at 21.7° due to inorganic SiO_2_ NPs on the film surface [54]. The neat PBAT/P(MMA-*co*-MA) blend exhibits reflection planes of (102), (012), and (113) for PBAT and the (002) peak for P(MMA-*co*-MA), respectively. Therefore, the PBAT/P(MMA-*co*-MA) blend forms a heterogeneous phase because of the poor miscibility or weak interactions between the two composites. These three peaks are found in the same positions throughout all PBAT/P(MMA-*co*-MA) blends, indicating that the SiO_2_ NPs alter the semi-crystal form of the PBAT/P(MMA-*co*-MA). These results also suggest that adding SiO_2_ as a filler has no noticeable effect on the semi-crystal form of PBAT/P(MMA-*co*-MA). The clean PBAT, P(MMA-*co*-MA) film, and PBAT/P(MMA-*co*-MA)–SiO_2_ composites with different percentages of (1.0 to 5.0 wt.%) SiO_2_ showed uniform surfaces because the viscosities of the respective film-forming solutions were suitable for casting films (Figure 5C). The PBAT/P(MMA-*co*-MA)–SiO_2_ composite film with 5.0 wt.% SiO_2_ (PBMS-3), however, had a rougher surface because of SiO_2_ agglomeration and air bubbles trapped in the casting solution. These flaws occurred because the viscosity of the PBAT solution with 5.0 wt.% SiO_2_ prevented air bubbles from exiting and the SiO_2_ content above the optimal value (~3.0 wt.%) enhanced PBAT and metal oxide (SiO_2_) interactions. Therefore, using SiO_2_ NPs, the effects of SiO_2_ on the barrier and mechanical characteristics of the PBAT/P(MMA-*co*-MA) blends were evaluated. The results were compared with the outcomes of the PBAT and PBAT/P(MMA-*co*-MA) composites.

**Figure 6:**
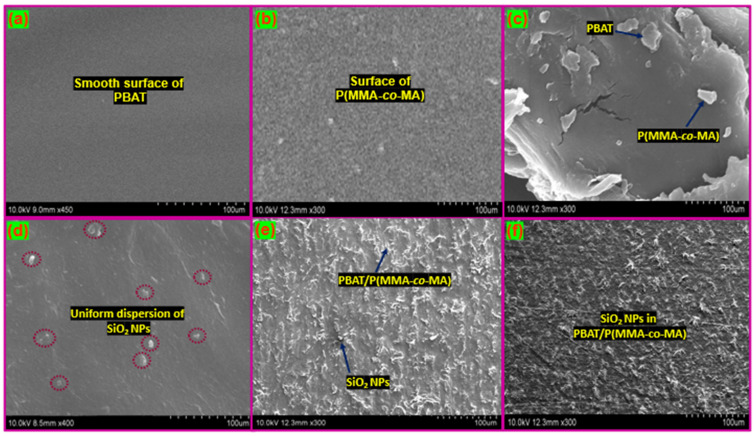
SEM images of the surfaces of PBAT/P(MMA-*co*-MA) blends and their composites: (**a**) PBAT, (**b**) P(MMA-*co*-MA), (**c**) PBAT/P(MMA-*co*-MA) blends, (**d**) 1.0, (**e**) 3.0, and (**f**) 5.0 wt.% of SiO_2_ NPs.

Figure 6 presents SEM images of the PBAT/P(MMA-*co*-MA)–SiO_2_ composites prepared with different wt.% loadings (SiO_2_). The figures show that the presence of SiO_2_ NPs in PBAT/P(MMA-*co*-MA) blends showed that the PBAT and P(MMA-*co*-MA) have smooth surfaces that face downward and that a rough surface was observed after loading a larger weight % of SiO_2_ NPs. Significant SiO_2_ aggregates dispersed in the PBAT/P(MMA-*co*-MA were observed in the SEM images, as illustrated by the red circles in Figure 6d. The SiO_2_ and the PBAT/P(MMA-*co*-MA) blend were visible, showing the poor compatibility of the two different polymers. Because of the similar composition ratios, PBAT/P(MMA-*co*-MA) (PBM-3) formed a continuous structure, as shown in Figure 6f. The green arrow in Figure 6e also shows that the formation of substantial amounts of SiO_2_ was induced by the ultra PBAT upon impact splitting. This played a major role in the high tensile strength of the ternary composites. The SEM image of the PBAT/P(MMA-*co*-MA)–SiO_2_ composite film was produced using the lowest amount of SiO_2_ (1.0 wt.%) (PBMS-1). In this image, the SiO_2_ in the PBAT/P(MMA-*co*-MA) blend is distributed evenly. SiO_2_ NPs agglomerate in the PBAT/P(MMA-*co*-MA) matrix as a result of the increased interactions among nanoparticles as the SiO_2_ concentration is increased. Based on these results, the C=O units of PBAT and P(MMA-*co*-MA) and SiO_2_ have strong bonding interactions in the developed ternary PBAT/P(MMA-*co*-MA)–SiO_2_ composites. SEM was used to establish that the SiO_2_ NPs and PBAT/P(MMA-*co*-MA) blends constituted a nanostructured composite material.

**Figure 7:**
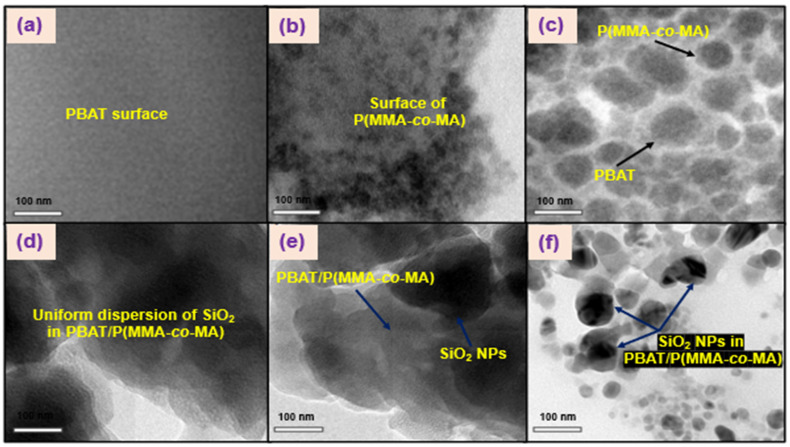
TEM images of PBAT/P(MMA-*co*-MA)–SiO_2_ ternary composites: (**a**) PBAT, (**b**) P(MMA-*co*-MA), (**c**) PBAT/P(MMA-*co*-MA) blends, (**d**) PBMS-2, (**e**) PBMS-2, and (**f**) PBMS-3.

The inner morphology of the PBAT/P(MMA-*co*-MA)–SiO_2_ composites was investigated using TEM and the results are shown in Figure 7. The SiO_2_ NPs were encapsulated in the PBAT/P(MMA-*co*-MA) matrix, as shown in these images. The transparent region of the images is a representation of the PBAT/P(MMA-*co*-MA) blend matrix. Figure 7a,b show the inner morphologies of PBAT and P(MMA-*co*-MA), and Figure 7c–e show a TEM image of PBAT/P(MMA-*co*-MA) blends, PBMS-1, PBMS-2, and PBMS-3 composites. The PBAT/P(MMA-*co*-MA) blend shows uneven morphology (small round structure and agglomerate merged) owing to the poor miscibility in the polymer blend, as seen in Figure 7c. The inner morphology of PBAT/P(MMA-*co*-MA)–SiO_2_ composites (Figure 7d–f) showed a distorted and smooth morphology owing to the bonding strength of the polymer matrix and SiO_2_ surface. Agglomerations of SiO_2_ NPs in the PBAT/P(MMA-*co*-MA) matrix are seen in Figure 7f. SiO_2_ had a high dispersion in the matrix of the PBAT/P(MMA-*co*-MA) blend. Independent of the SiO_2_ concentration, all the composites showed a dispersed morphology of SiO_2_ when evaluating how the SiO_2_ of the PBAT and P(MMA-*co*-MA) influences the morphology of the systems. The composites of PBAT/P(MMA-*co*-MA)–SiO_2_ showed fewer interactions and inner morphology according to the TEM of PBMS-3. SiO_2_ is a suitable reinforcing filler and compatibilizer in PBAT/P(MMA-*co*-MA) blends, as shown by TEM.

### 3.4. Thermal Properties of PBAT/P(MMA-co-MA)–SiO_2_ Composite Films

#### 3.4.1. Thermogravimetric Analysis (TGA)

**Figure 8:**
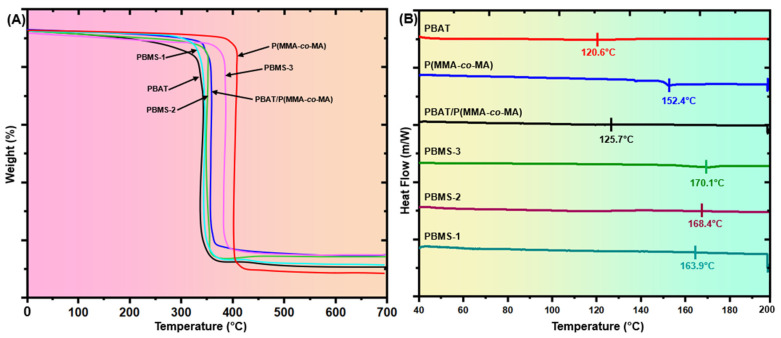
(**A**) TGA and (**B**) DSC curves of PBAT, P(MMA-*co*-MA), PBAT/P(MMA-*co*-MA), and PBAT/P(MMA-*co*-MA)–SiO_2_ composites.

Figure 8A shows the TGA curves of PBAT/P(MMA-*co*-MA) composites with different SiO_2_ NPs concentrations. Table 2 lists the thermal parameters. The two-step thermal degradation behavior was observed in all PBAT/P(MMA-*co*-MA)–SiO_2_ composites. The weight loss at 350 and 450 °C was attributed to the thermal degradation of P(MMA-*co*-MA). The weight loss at temperatures above 342.3 °C was caused mostly by the degradation of PBAT. The least stable blend of PBAT and P(MMA-*co*-MA) was without SiO_2_ NPs. The temperature of the initial weight loss of the PBAT/P(MMA-*co*-MA) blend was only 382.5 °C, and the temperature of the final weight loss was only 403.1 °C. The thermal stability of the composites improved significantly as the SiO_2_ NPs content increased. SiO_2_ could act as a barrier inside the composites, slowing the diffusion of the decomposition of PBAT and P(MMA-*co*-MA) while limiting oxidative degradation and increasing the thermal stability of the composite materials. PBAT/P(MMA-*co*-MA)–SiO_2_ (PBMS-3) composites showed the highest thermal stability of the PBAT/P(MMA-*co*-MA) composites containing different amounts of SiO_2_, whereas PBAT/P(MMA-*co*-MA)–SiO_2_ (PBMS-1) exhibited the lowest thermal stability. SiO_2_ can graft onto polymer chains owing to the ability of SiO_2_ NPs to interact with P(MMA-*co*-MA) or PBAT. These interactions improve their compatibility and dispersibility in polymer matrices and their physical barrier effect within the matrix. Another factor is the effectiveness of the reaction between the PBAT/P(MMA-*co*-MA) and SiO_2_. The PBAT/P(MMA-*co*-MA)–SiO_2_ (PBMS-3) composites showed higher thermal stability.

#### 3.4.2. Differential Scanning Calorimetry (DSC)

The melting temperature (Tm) and glass transition temperature (Tg) were measured for PBAT/P(MMA-*co*-MA)–SiO_2_ composites using DSC to evaluate the thermal stability of film components. Figure 8B shows the DSC thermograms of PBAT/P(MMA-*co*-MA) and PBAT/P(MMA-*co*-MA)–SiO_2_ composites. Table 2 lists the relevant DSC data. The PBAT and P(MMA-*co*-MA) film had a T_g_ of 49.3 and 53.9 °C, respectively. In contrast, the SiO_2_-induced PBAT/P(MMA-*co*-MA) composites showed a T_g_ (52.1 to 65.2 °C) with an increase in SiO_2_ content, indicating good compatibility of the components of the (PBMS) composites. Furthermore, the melting temperature (Tm) of the PBAT/P(MMA-*co*-MA) blend film was 125.7 °C, and the PBAT/P(MMA-*co*-MA)–SiO_2_ composites showed a single melting temperature (Tm) that increased from 163.9 to 170.1 °C as the SiO_2_ content was increased from 1.0 to 5.0 wt.%. The T_g_ and Tm values in the composites of PBAT/P(MMA-*co*-MA)–SiO_2_ suggest that the ternary components interact to generate good comparability among PBAT, P(MMA-*co*-MA), and SiO_2_. The melting temperature values of composites increased growth in the SiO_2_ from 1.0 to 5.0 wt.%, showing that SiO_2_ incorporation enhanced PBAT crystallization. The addition of 1.0 wt.% SiO_2_ reduced the crystallinity of the PBMS-1 film significantly compared to the PBAT/P(MMA-*co*-MA) blend. By contrast, the addition of 3.0 and 5.0 wt.% SiO_2_ significantly increased the crystallinity of the composite films PBMS-2 and PBMS-3.

### 3.5. Mechanical Strength of PBAT/P(MMA-co-MA)–SiO_2_ Composite Films

**Figure 9:**
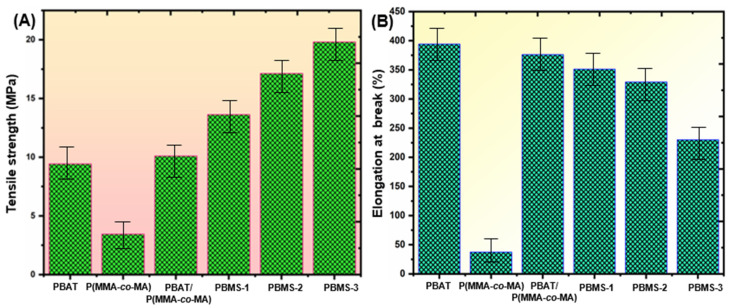
Mechanical characterizations of PBAT/P(MMA-*co*-MA)–SiO_2_ composites: (**A**) tensile strength (MPa) and (**B**) elongation at break (%). Error bars represent ±5.01 standard errors.

Inorganic nanofillers are frequently incorporated into polymers to strengthen the mechanical characteristics of the resulting composites. In particular, the materials used to package food should be strong and rigid enough to support themselves and resist handling damage. The tensile and elongation at break (EB) of PBAT/P(MMA-*co*-MA)–SiO_2_ ternary composites are higher than those of PBAT and PBAT/P(MMA-*co*-MA) blends, as shown in Figure 9. The tensile strength of the PBAT/P(MMA-*co*-MA)–SiO_2_ ternary composites was SiO_2_-dependent, as shown in Figure 9A. The tensile strength of the PBAT/P(MMA-*co*-MA)–SiO_2_ composites increased as the SiO_2_ concentration increased. This could be because of the strong bonding between the PBAT/P(MMA-*co*-MA) blends and SiO_2_ interfacial adhesion. The elongation at break of the ternary composites decreased from 394.28 to 230.12% as the SiO_2_ loading increased (Figure 9B). This is because SiO_2_ is blended into PBAT/P(MMA-*co*-MA)*,* decreasing the van der Waals strength among PBAT/P(MMA-*co*-MA) blend and SiO_2_ NPs.

The tensile and EB of neat PBAT matrix (9.43 MPa and 394.2%, respectively) and PBAT/P(MMA-*co*-MA) blends (10.10 MPa and 376.1%, respectively) were similar, resulting from less attraction to the PBAT/P(MMA-*co*-MA). On the other hand, a noteworthy improvement in tensile strength (19.81 MPa, a 230.1% increase) was facilitated by the high content of SiO_2_ NPs (5.0 wt.%). The enhancement in tensile strength appears to result from an H-bond connection between the ester bond of the PBAT/P(MMA-*co*-MA) matrix and the Si–O group of the SiO_2_ NPs. Because H-bonds and van der Waals forces are produced in PBAT/P(MMA-*co*-MA)–SiO_2_ (PBMS-3) composites, their mechanical properties are improved, resulting in a strong interaction between them. The tensile and EB of PBMS-3 composites were superior to those of other PBMS films because of the surface function. With the addition of SiO_2_, the lower percentage of the elongation at break from 34.28% to 18.01%, the brittle interaction between the SiO_2_ and the PBAT/P(MMA-*co*-MA) blended material to become less flexible could be responsible.

### 3.6. Water Contact Angle

**Figure 10:**
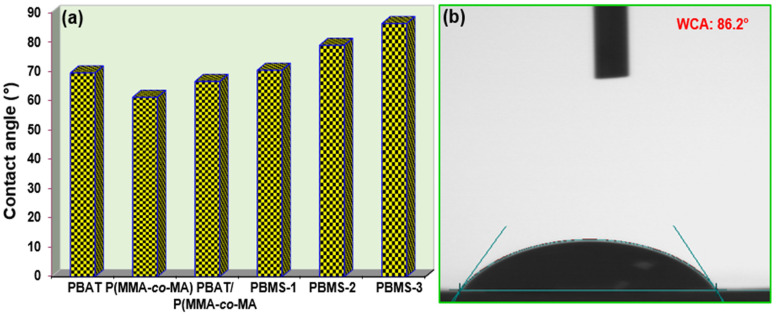
Contact angle of composite film samples (**a**) and PBAT/P(MMA-*co*-MA)–SiO_2_ (5.0 wt.%) image (**b**).

The water contact angles of the composites were measured. The film area appeared to be round because of the sudden change in the film after droplets were placed on the surface for every film in Figure 10. Figure 10 shows the pure PBAT film, revealing a contact angle of =69.4°, indicating its hydrophobicity. The pure PBAT film has a contact angle consistent with current research [55,56]. In contrast to P(MMA-*co*-MA), hydrophobicity was confirmed with a contact angle of =61.1°. The hydrophobic materials on the surface of the film of PBMS-3 have a contact angle of 86.2°. SiO_2_ addition resulted in a higher contact angle than P(MMA-*co*-MA) (25.1°) and a lower angle than PBAT films (16.8°) after addition. A comparison of PBAT/P(MMA-*co*-MA) with the PBMS-3 film showed that the contact angle of these films was lower, and the affinity for water increased. As SiO_2_ is incorporated into PBAT and P(MMA-*co*-MA) blend, the polar groups can give a contact angle that lowers the upward aspect.

### 3.7. Barrier Properties

Food packaging materials should have barrier properties for oxygen, or water may accelerate food spoilage in packing. It is essential to have low OTR and WVTR [57]. Table 3 lists the OTR and WVTR values of composite films produced by PBAT/P(MMA-*co*-MA)–SiO_2_ with all these characteristics. The NP content caused a decrease in the OTR and WVTR values, indicating that the PBAT/P(MMA-*co*-MA)–SiO_2_ ternary composites have good barrier properties. Previous studies reported activities in the OTR and WVTR for SiO_2_-reinforced PBAT [58]. Water and oxygen molecules can travel through PBAT films completely free of impurities. On the other hand, these small molecules in the PBAT/P(MMA-*co*-MA)–SiO_2_ composites encounter a long and circuitous pathway because of the requirement to migrate over or through the interfaces of impassable SiO_2_.

As a result, the WVP and OTR values are decreased when SiO_2_ NPs are introduced. WVTR must be reduced to reduce water transfer between the packing material and the material used for packaged foods. According to Table 3, the occurrence of SiO_2_ NP content leads the oxygen transmission rate values ranging from 1137.2 to 318.9 (cc m^−2^ per 24 h). SiO_2_ incorporation into the PBAT/P(MMA-*co*-MA) film reduced the OTR values of the ternary composites. The WVTR of the PBAT/P(MMA-*co*-MA) blend film was 82.9 (g m^−2^ per 24 h), whereas the WVTR of the PBAT/P(MMA-*co*-MA)–SiO_2_ composites was lowered by the addition of SiO_2_ NPs to 26.3 (g m^−2^ per 24 h). The WVTR of neat PBAT is 127.1 (g m^−2^ per 24 h). In the ternary PBAT/P(MMA-*co*-MA)–SiO_2_ composites, there was a modest reduction in WVTR in the event of PBAT because of a decrease in hydrogen bonding with PBAT/P(MMA-*co*-MA) and the base film. This was attributed to the water-available absorption area. Incorporating 1.0 wt.% SiO_2_ NPs significantly decreased the WVTR value to 63.2 (g m^−2^ per 24 h). The permeability decreased dramatically at the highest concentration of SiO_2_ NPs (5.0 wt.%). SiO_2_ NP loading decreased the WVTR by allowing water vapor to flow in zigzag pathways through the dispersed nanoparticles. On the other hand, the SiO_2_ NPs tended to band together at higher concentrations, which decreased the effective content that promoted WVTR.

### 3.8. Antimicrobial Activities of PBAT/P(MMA-co-MA)–SiO_2_ Composites

**Figure 11:**
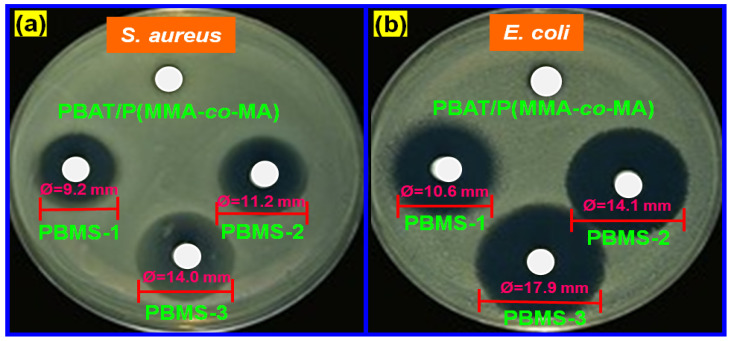
Antimicrobial test results of PBAT/P(MMA-*co*-MA) and their composite films against (**a**) *S. aureus* and (**b**) *E. coli*.

The antimicrobial properties of the metal oxide nanoparticles were good, and their reinforcement into the polymeric matrix significantly improved the antimicrobial property of the film. The zone-of-inhibition method was used to evaluate the antimicrobial property of PBAT/P(MMA-*co*-MA)–SiO_2_ composite films. Figure 11 shows the results, and Table 4 lists the diameters of the film inhibition zones after calculating their specimen size. Th PBAT, P(MMA-*co*-MA), and PBAT/P(MMA-*co*-MA) films had no antimicrobial activities. The PBAT/P(MMA-*co*-MA) film loaded with a specific amount of SiO_2_ will induce a zone of inhibition for microorganisms that are pathogenic to food. In tests against *S. aureus* and *E. coli* at concentrations of 1.0, 3.0, and 5.0 wt.% SiO_2_, the PBAT/P(MMA-*co*-MA)–SiO_2_ composites showed good antimicrobial activities compared to the PBAT and PBAT/P(MMA-*co*-MA) blends. The PBAT/P(MMA-*co*-MA)–SiO_2_ ternary composites showed good antimicrobial characteristics when the SiO_2_ NP concentration was as low as 1.0 wt.% (minimum (*E. coli*) 10.6 mm; (*S. aureus*) 9.2 mm), whereas the maximum ((*E. coli*) 17.9 mm; (*S. aureus*) 14.0 mm) was observed when the SiO_2_ concentration was 5.0 wt.%. Compared to the PBAT composites, which are reported elsewhere, the antimicrobial properties were stronger [59]. In contrast to the report of a film incorporating SiO_2_, the SiO_2_ NP-incorporated high-antimicrobial-diameter film had a significantly higher zone of inhibition than the PBAT for the same composition. Increasing the SiO_2_ concentration expanded the inhibition zone of the SiO_2_-incorporated ternary composite films, showing that the PBAT/P(MMA-*co*-MA) film can function as a film that is active against both pathogens. *E. coli* was negatively charged and had less surface area than *S. aureus*. Furthermore, based on the observations, the PBMS-3 composite film exhibited strong antimicrobial activity against *S. aureus* and *E. coli* than the other films [60].

## 4. Conclusions

A poly(methyl methacrylate-*co*-maleic anhydride) P(MMA-*co*-MA) copolymer was produced by radical polymerization. Solution casting was used to produce PBAT/P(MMA-*co*-MA) composite films with different SiO_2_ concentrations. The addition of SiO_2_ had a major effect on the mechanical, H_2_O and O_2_ barrier properties, thermal properties, and antimicrobial activity characteristics of the film. The tensile strength of the PBAT/P(MMA-*co*-MA) film was enhanced by the addition of SiO_2_. The inclusion of SiO_2_ improved the miscibility between PBAT and P(MMA-*co*-MA), according to the SEM and TEM results. The PBMS-3 composite film enhanced the elongation at break and tensile strength after adding SiO_2_ to the PBAT/P(MMA-*co*-MA), allowing it to be studied as a structurally steady food packaging. Compared to PBAT/P(MMA-*co*-MA), the WVTR and OTR were much lower for the PBAT/P(MMA-*co*-MA)–SiO_2_ composite films. Furthermore, SiO_2_ produced PBAT/P(MMA-*co*-MA) materials that were more hydrophobic by increasing the contact angle. The water contact angle of PBAT/P(MMA-*co*-MA) was improved from 61.1 to 86.2 by introducing additional SiO_2_ NPs, which enhanced the hydrophobicity. SiO_2_-incorporated PBAT/P(MMA-*co*-MA) composite films showed effective antimicrobial activity against *S. aureus* and *E. coli*. The results suggest that the PBAT/P(MMA-*co*-MA)–SiO_2_ composites can be used as materials for food packaging, which minimizes the microbiological load and extends the shelf life of packaged foods.

## Figures and Tables

**Table 1:** Material formulation in blends and composite film preparation.

Samples	PBAT (wt.%)	P(MMA-*co*-MA) (wt.%)	SiO_2_ NPs (wt.%)
PBAT	100.0	-	-
P(MMA-*co*-MA)	-	100.0	-
PBAT/P(MMA-*co*-MA)	70.0	30.0	-
PBMS-1	70.0	30.0	1.0
PBMS-2	70.0	30.0	3.0
PBMS-3	70.0	30.0	5.0

Abbreviations: PBAT, poly(butylene adipate-*co*-terephthalate); P(MMA-*co*-MA), Poly(methyl methacrylate-*co*-maleic anhydride); PBMS, PBAT/P(MMA-*co*-MA)–SiO_2_; SiO_2_, silicon dioxide.

**Table 2:** TGA and DSC results of PBAT/P(MMA-*co*-MA) blends and their composites.

Samples	TGA			DSC	
	Initial Degradation Temperature (°C) ^a^	Final Degradation Temperature (°C) ^b^	Ash Content (%) ^c^	Tg (°C)	Tm (°C)
PBAT	322.3	358.2	3.59	49.3	120.6
P(MMA-*co*-MA)	395.0	420.6	2.56	53.9	152.4
PBAT/P(MMA-*co*-MA)	368.4	385.2	1.68	56.0	125.7
PBMS-1	348.1	359.4	1.13	52.1	163.9
PBMS-2	364.9	379.0	1.41	60.7	168.4
PBMS-3	382.5	403.1	2.06	65.2	170.1

^a^ Temperature at which the initial mass loss was recorded. ^b^ Temperature at which the final mass loss was recorded. ^c^ Mass percentage of material remaining after TGA at a maximum temperature of 700 °C.

**Table 3:** Barrier properties of PBAT/P(MMA-*co*-MA)–SiO_2_ composite films.

Samples	Oxygen Transmission Rate, (cc m^−2^ Per 24 h)	Water Vapor Transmission Rate, (g m^−2^ Per 24 h)
PBAT	1137.2 ± 2.5 ^a^	127.1 ± 2.9 ^b^
P(MMA-*co*-MA)	860.2 ± 3.0 ^a^	41.0 ± 2.2 ^a^
PBAT/P(MMA-*co*-MA)	1095.6 ± 2.0 ^c^	82.9 ± 3.1 ^c^
PBMS-1	824.1 ± 3.4 ^a^	63.2 ± 2.7 ^c^
PBMS-2	589.3 ± 2.7 ^b^	41.0 ± 3.0 ^a^
PBMS-3	318.9 ± 2.0 ^c^	26.3 ± 2.5 ^a^

a–c: Different letters within the same column indicate significant differences among the film samples (*p* < 0.05).

**Table 4:** Antimicrobial activity test values of the PBAT/P(MMA-*co*-MA) and PBAT/P(MMA-*co*-MA)–SiO_2_ composites against *S. aureus* and *E. coli*.

Strain	Zone of Inhibition in (mm)			
	PBAT/P(MMA-*co*-MA)	PBMS-1	PBMS-2	PBMS-3
*S. aureus*	-	9.2 ± 3.84 ^c^	11.2 ± 1.85 ^a^	14.0 ± 2.63 ^b^
*E. coli*	-	10.6 ± 2.35 ^b^	14.1 ± 3.31 ^c^	17.9 ± 2.56 ^c^

Results are quoted as the mean ± standard deviation of three replicates. a–c: Different letters within the same column indicate significant differences among the film samples (*p* < 0.05).

## Data Availability

Not applicable.
